# Numerical Fatigue Crack Growth on Compact Tension Specimens under Mode I and Mixed-Mode (I+II) Loading

**DOI:** 10.3390/ma17184570

**Published:** 2024-09-18

**Authors:** Rui F. Martins, José Xavier, João Caldeira

**Affiliations:** 1 UNIDEMI, Department of Mechanical and Industrial Engineering, NOVA School of Science and Technology, Universidade NOVA de Lisboa, Campus de Caparica, 2829-516 Caparica, Portugal; jmc.xavier@fct.unl.pt (J.X.); jr.caldeira@campus.fct.unl.pt (J.C.); 2 Intelligent Systems Associate Laboratory (LASI), 4800-058 Guimarães, Portugal

**Keywords:** fracture mechanics, mode I and mixed-mode (I+II) loading, Abaqus^®^, XFEM, AISI 316L, fatigue life prediction

## Abstract

This study focused on standard Compact Tension (CT) specimens and two loading modes during the numerical analyses carried out, namely: pure mode I and mixed-mode loading (Modes I+II). Numerical stress intensity factors, K_I_, were calculated using Abaqus^®^ 2022 and compared with those given analytically under pure mode I loading, showing very good agreement. Additionally, K_I_, K_II_, and K_III_ results obtained from Abaqus^®^ were presented for mixed-mode loading, analyzing crack growth and variation through the thickness of the CT specimen. Moreover, fatigue crack growth simulations under mode I loading were conducted on standard CT specimens using the Extended Finite Element Method (XFEM) and the Paris Law parameters of an AISI 316L stainless steel. It was shown that XFEM effectively determines crack propagation direction and growth, provided that an appropriate mesh is implemented.

## 1. Introduction

Human activity has been interested in structural integrity since its earliest days, as it reflects the quality of the design and manufacture, as well as the equipment’s ability to function within its intended service life. Moreover, failure in mechanical equipment can have severe human and economic consequences. Therefore, achieving integrity in the service requires careful analysis of design, construction, and use stages, including material selection, manufacturing, assembly, quality control, and operational monitoring.

Hence, understanding the mechanical fatigue behavior of materials is of great importance, and the possibility of reliably predicting the useful life of materials allows for the development of more reliable and, naturally, safer components. However, predicting the fatigue life of equipment is not a simple task due to the intrinsic complexity of the fatigue process. In fact, according to the ASTM E1823-2002 standard [1], fatigue is defined as a process of permanent, progressive, and localized structural alteration that occurs in a material subjected to conditions that produce dynamic stresses or extensions at one point or at several points. This alteration can culminate in the propagation of cracks or in a fracture after a sufficient number of fluctuations (cycles). At the same time, fracture mechanics, in particular Linear Elastic Fracture Mechanics (LEFM), play a significant role in preventing structural failures, and, when applied correctly in association with fatigue fundamentals, prevent failure, since designers are supported by rational analysis and not just trial and error [2].

A parameter linked to fracture mechanics that controls the propagation of cracks in components subjected to external forces is the stress intensity factor, K, which depends on the geometry of the component, the size, shape, and location of the crack, and the intensity and mode of loading applied. There are three loading modes (I, II, and III), each associated with a specific stress intensity factor (K_I_, K_II_, K_III_) (Equation (1)). Mode I (opening mode) is characterized by a load applied in a direction perpendicular to the crack plane, resulting in normal tensile stresses at the end of the crack. In this mode, crack propagation always occurs in the direction perpendicular to the loading plane. Mode II (“In-Plane Shear”) applies a load in the direction parallel to the crack surface, promoting shear stresses at its end. Mode III (“Out-of-Plane Shear”) is defined by loading in the direction parallel to both the surface and the front of the crack, which also causes shear stresses at the end of the crack.

(1) $$\sigma_{ij}\left(r,\theta \right)=\frac{K_{I,II,III}}{\sqrt{2\pi r}}\cdot f_{ij}\left(\theta \right)+A_{1}{\left(\frac{r}{a}\right)}^{0}\cdot f_{ij}\left(\theta \right)+A_{2}{\left(\frac{r}{a}\right)}^{1/2}\cdot f_{2ij}\left(\theta \right)+\ldots$$

Once the three basic loading modes coexist, temporarily or permanently, stress intensity factors of the K_I_, K_II_, K_III_ types will be present. For pure mode I loading, there are defined criteria for the propagation of fatigue cracks, and it can be stated that the crack will propagate once the stress intensity factor, ∆K_I_, is greater than the threshold stress intensity factor, ∆K_Ith_. Moreover, if the value of K_I_^max^ exceeds the mode I fracture toughness, K_Ic_, the crack will propagate suddenly and unstably. These properties can be obtained experimentally and are available for a wide variety of materials. However, using these criteria for situations involving mixed loading of any kind, whether planar (mode I+II) or spatial (mode I+II+III), the results obtained would not be conservative, since K_II_ and K_III_ will also contribute to a higher equivalent stress intensity factor [3,4]. 

In addition, few existing results on mixed-mode propagation exist even though many engineering cases presuppose it, and the possibility of using numerical simulation overcomes the difficulties involved in carrying out experimental tests of this nature. Zhu [5], for instance, simulated the growth mechanism of fatigue cracks in a bridge under the load of vehicles using XFEM from Abaqus^®^, and Mutoh et al. [6] numerically simulated fretting fatigue crack initiation and propagation also using Abaqus^®^. Moreover, in [7], utilizing the commercial software Abaqus^®^, the fatigue crack path in large-scale marine structures was simulated, and the corresponding fatigue life was computed. Single crack propagation, as well as multiple cracks in case of simultaneous but uneven growth, was addressed. Additionally, Jie et al. [8] simulated residual stress influence on the fatigue crack propagation of CFRP-strengthened welded joints using XFEM.

Other works that did not involve determining the direction and size of the propagated crack but rather determined the values of the stress intensity factors in mode I and/or mixed mode using Abaqus can be found in greater numbers [9,10,11,12,13,14]. Moreover, numerical analyses carried out in Abaqus are frequently combined with other software, such as Zencrack (Zentech International Ltd., London, U.K.) or Franc2D/3D (Cornell University, New York, U.S.A.), to perform numerical fatigue crack propagation simulations [15,16,17,18,19].

In this way, the work carried out aims to demonstrate that numerical simulation, based on the experimental data of the materials, can approximate the computational values to the experimental values of the fatigue life of CT specimens. This can be followed by the simulation of crack propagation in real engineering components with high geometric complexity and subject to spatial loading in order to estimate accurate fatigue life that could guarantee structural integrity and serve as an aid to periodic inspections and maintenance procedures.

In the study herein presented, it was possible to simulate the propagation of cracks, static and direct cyclic, in a Compact Tension (CT) specimen under mode I and mixed mode (I+II) using the Abaqus^®^ software (2022, Dassault Systèmes: Vélizy-Villacoublay, France) and the Extended Finite Element Method (XFEM). Hence, static analyses allowed us to obtain the direction of crack propagation and calculate the stress intensity factors for several different crack lengths, and along the CT specimen’s thickness, for mode I and mixed-mode loading (mode I+II). In addition, in the case of the simulation of fatigue crack propagation (direct cyclic), based on the Paris Law, the aim was to calculate the number of cycles for specific crack lengths using dynamic (cyclic) analysis.

## 2. Materials and Methods

**Figure 1:**
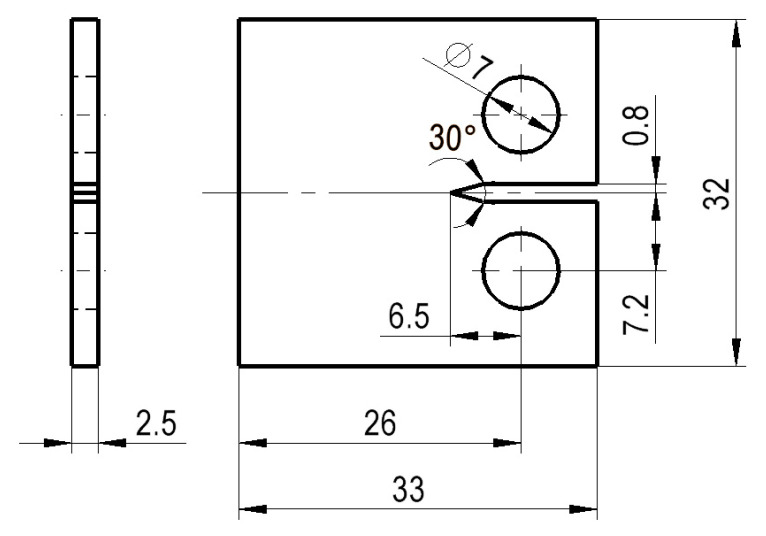
Compact Tension (CT) specimen, with a thickness of 2.5 mm (unit: millimeters).

The dimensions of the CT specimen utilized in the numerical simulations are shown in Figure 1 and correspond to those reported in [20,21], with a thickness of 2.5 mm. The specimen contains a notch in its geometry from which the crack propagates upon the application of a load. In the case of mode I loading, the analytical solution for the stress intensity factor, K_I_, is given by Equation (2) [22]:

(2) $$K_{I}=\frac{P}{B\sqrt{W}}f\left(\frac{a}{W}\right)\;f\left(\frac{a}{W}\right)=\frac{2+\frac{a}{W}}{{\left(1-\frac{a}{W}\right)}^{\frac{3}{2}}}\left[0.886+4.64\left(\frac{a}{w}\right)-13.32{\left(\frac{a}{w}\right)}^{2}+14.72{\left(\frac{a}{w}\right)}^{3}-5.6{\left(\frac{a}{w}\right)}^{4}\right]$$

where *P* is the applied load, *B* is the specimen thickness, *W* is the effective width, and *a* is the crack length. The specimens are made of an AISI 316L austenitic stainless steel with the chemical composition presented in Table 1 and the mechanical properties given in Table 2 [20,21].

**Figure 2:**
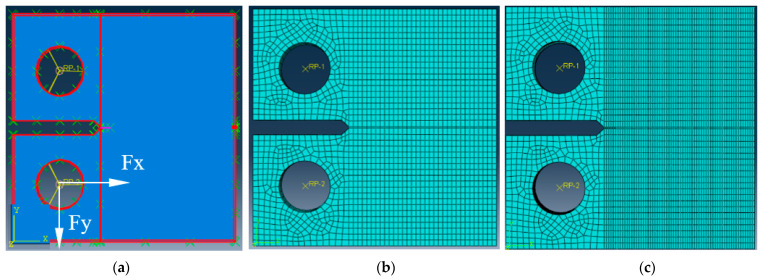
Compact Tension (CT) specimen. (**a**) With specified pre-crack, boundary conditions and forces; (**b**) finite element mesh used during fracture simulation tests (mode I and mode I+II); (**c**) finite element mesh used during fatigue crack growth (FCG) simulation tests.

A through-thickness surface pre-crack with a 1 mm length was placed at the tip of the modelled notch (Figure 2a), and a linear elastic, homogeneous, and isotropic material model was considered (Table 2).

To define the boundary conditions, two reference points are established at the center of each of the specimen’s holes and midway through its thickness, as shown in Figure 2b. These pivot points are used to apply the load at the internal surfaces of both holes, thereby replicating the experimental setup that employed two pins to transfer the fracture loading. The points for null displacement/rotation and application of forces—*Fy*, *Fx*—equal to 2000 N at pin holes, as well as crack location, growth, and the use of XFEM, are shown in Figure 2a.

In addition to the initial step, where boundary conditions were defined, the subsequent step was defined as static/general or direct cyclic for fracture or fatigue crack growth (FCG) simulations, respectively. Five contours were considered to calculate the stress intensity factors at each node placed at the crack tip, along the thickness of the CT specimen, at different crack lengths, through the determination of J-integral values. Moreover, crack growth was defined according to the maximum energy release rate criterion, G_c_.

A C3D8R linear finite element with eighth nodes and reduced integration was used to create the meshes, namely with 7760 elements and 10,365 nodes for the fracture simulation tests (mode I and mode I+II) (Figure 2b) and with 48,780 elements and 54,672 nodes for FCG (Figure 2c). These correspond to a finite element edge size ahead of the crack tip, equal to 0.6 mm and 0.34 mm, respectively. The number of elements placed across the thickness of the specimen was equal to 4 and 15, which corresponded to the fracture and FCG tests, respectively, and a finite element edge size of 0.63 mm and 0.17 mm, respectively. As the analysis of crack propagation under fatigue is more complex than that of fracture toughness tests, the mesh generated for the FCG simulations was significantly more refined to ensure accurate propagation. Static and direct cyclic analyses were carried out to simulate the fracture and FCG tests, respectively.

For the fatigue crack growth simulation, a load ratio, *R*, of 0.1 was defined. To analyze crack propagation under fatigue, it is necessary to define a cyclic loading pattern to be applied to the specimen. This loading is characterized by multiple cycles of loading and unloading, with a load ratio, *R*, of 0.1. In Abaqus, this cyclic load was defined using the Amplitude Tabular functionality. The input data for this functionality consist of a load multiplier factor associated with three time points. Given that the applied load is 2000 N, the data entered to achieve the desired loading cycle are three amplitudes of 0.1, 1, and 0.1 at times 0, 0.5, and 1, respectively.

The FCG simulations assumed that a mode I crack propagates according to the Paris law (Regime II) as a function of the energy release rate parameter Δ*G* (Equation (3)), rather than the conventional form that involves the Δ*K_I_* stress intensity factor range, as described by

(3) $$\frac{da}{dN}=c_{3}\cdot ∆G^{c_{4}}$$

Moreover, the calculations involving an equivalent G_equiv_ value resulting from the combination of G_I_, G_II_, and G_III_ were considered using the Benzeggagh–Kenane (BK) Law (Equation (4)) [23]:

(4) $${G_{equiv}}_{c}={G_{I}}_{c}+\left({G_{II}}_{c}-{G_{I}}_{c}\right)\cdot {\left(\frac{G_{II}+G_{III}}{G_{I}+G_{II}+G_{III}}\right)}^{\eta }$$

However, *G_II_* and *G_III_* were residual for the FCG case considered and the *G_IIc_* critical value was set to zero. In addition, the Paris Law was implemented in Abaqus^®^ through the following line of code:

fracture criterion, type = fatigue, mixed-mode behavior = BK, tolerance = tol

*c*_1_, *c*_2_, *c*_3_, *r*_1_, *r*_2_,* G_Ic_*, *G_IIc_*, *G_IIIc_*, *η*

The values of c_1_ and c_2_ are 0.5 and −0.1, respectively, which correspond to the values of a typical steel fatigue crack initiation [24]; r_1_ and r_2_ were set to 0.01 and 0.85, respectively, and correspond to the ratio *G_threshold_*/*G_Ic_* and the ratio of *G* at the end of Regime II and *G_Ic_*. The exponent η was set to 1, which is the default value used by Abaqus^®^, and *G_IIc_*, *G_IIIc_*,  are equal to zero since propagation occurred under mode I loading. The remaining c_3_, c_4_, and *G_Ic_* values (Equations (5)–(7)) were calculated using the values of C, m from the Paris Law and *K_Ic_* determined experimentally by Martins and Branco [21] (Table 3), and are given as follows:

c_4_ = m/2 = 1.67 (5)

c_3_ = C × E^c4^ = 1.04 × 10^−9^ × (200 × 10^3^)^1.67^ = 0.741 (6)

(7) $$G_{I_{c}}=\frac{K_{IC}^{2}}{E}=50,000\;J/m^{2}$$

## 3. Results and Discussion

### 3.1. Fracture Mechanics (Static/General) under Mode I Loading (Fy = 2000 N)

**Figure 3:**
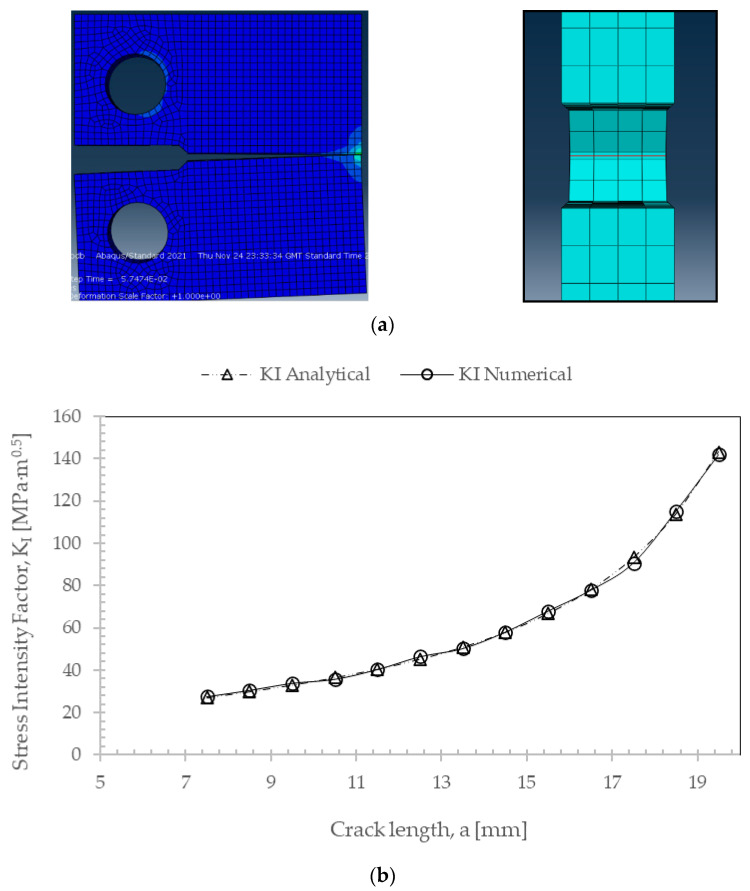
(**a**) Frontal view of Compact Tension (CT) specimen under mode I loading with horizontal crack propagation (*θ* = 0°) and lateral view of the CT specimen with finite elements placed across the thickness of the specimen. (**b**) Stress intensity factor values, K_I_ [MPa·m^0.5^], with increasing crack length, a [unit: mm].

The numerical results obtained for K_I_ ranged from 27.1 MPa·m^0.5^ to 90.7 MPa·m^0.5^ in the validity domain and were compared with those obtained through Equation (2) (Figure 3). A good agreement was achieved between numerical and analytical results (Table 4), with the numerical results being slightly higher than the analytical, with a deviation lower than 2.5%. It is also perceived that almost constant values for K_I_ across the thickness of the specimen were obtained (Table 5). Moreover, when a straight crack is loaded in mode I, the crack tip is expected to grow in the plane of the crack (*θ* = 0°).

### 3.2. Fracture Mechanics (Static/General) under Mixed-Mode Loading (Fy, Fx = 2000 N)

**Figure 4:**
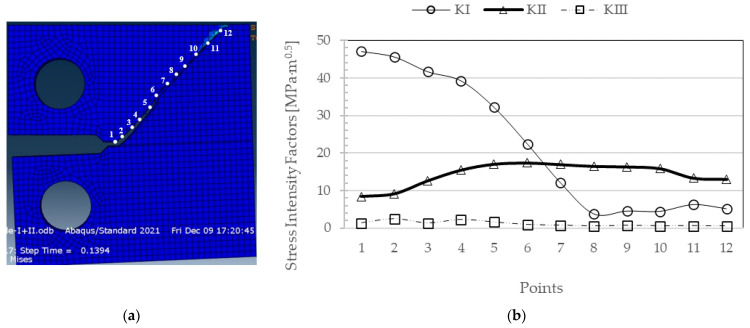
(**a**) Crack path propagation in a CT specimen under mixed-mode loading (I+II) along 12 points; (**b**) stress intensity factor values K_I_, K_II_, and K_III_ [MPa·m^0.5^] with increasing crack length.

In this loading mode case, the crack path is not obvious, and XFEM numerical simulation becomes essential. Therefore, the results are presented as a function of the data points along the crack preparation path. The results obtained are presented in Figure 4; twelve points were considered along the crack path to monitor the stress intensity factors K_I_, K_II_, and K_III_ (Figure 4).

**Figure 5:**
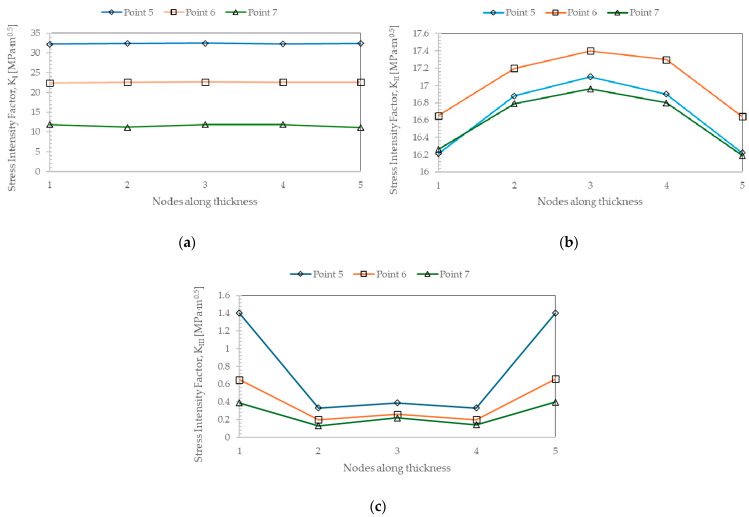
Stress intensity factor values along the specimen’s thickness at points 5, 6, and 7 under mixed-mode loading (I+II): (**a**) K_I_; (**b**) K_II_; (**c**) K_III_.

The results show that the maximum value of K_I_ is recorded right at the pre-crack (point 1) and decreases to its minimum value, corresponding to the crack length identified by point 8, remaining almost unchanged until the end of crack growth. Concerning K_II_, after the pre-crack, there is an increase to its maximum value at point 7, after which there is a slight decrease until the end of crack propagation. The values for K_III_ are residual, with no significant variations, and the highest values are found at the first points of crack propagation. Regarding the evolution of the K_I_, K_II_, and K_III_ values along the thickness of the specimen, and in the same way as the previous analysis, the five nodes along the thickness of the specimen at points 5, 6, and 7 were analyzed. The results are shown in Figure 5. In fact, K_I_ values remain constant throughout the thickness of the specimen. On the other hand, although the variations are slight, the K_II_ values peak at the center of the specimen. As for the K_III_ values, they show symmetry from the center of the specimen to the side surfaces, with a maximum near the latter. The only difference is that the minimum is not precisely in the specimen’s center but at nodes 2 and 4, with a slight increase in the center of the specimen compared to the nodes mentioned earlier.

### 3.3. Fatigue Crack Growth Simulation (Direct Cyclic) under Mode I Loading (Fy = 2000 N)

**Figure 6:**
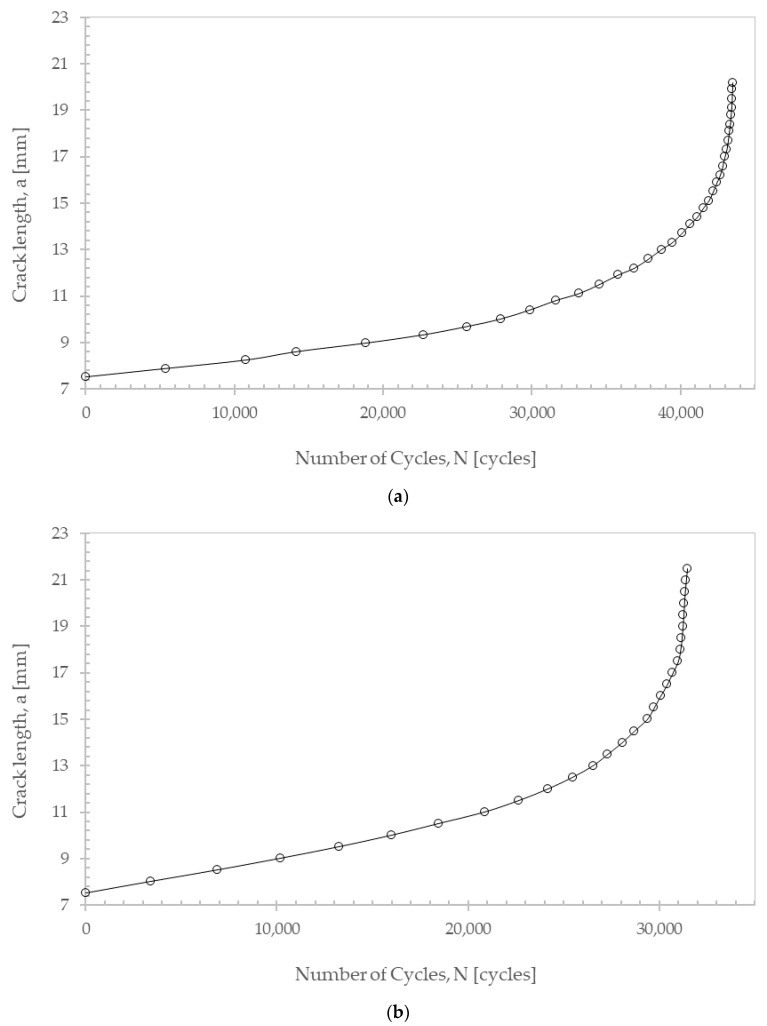
Crack length versus number of propagated cycles (**a**) for the 55-element mesh along the propagation length; (**b**) for the 40-element mesh along the propagation length.

**Figure 7:**
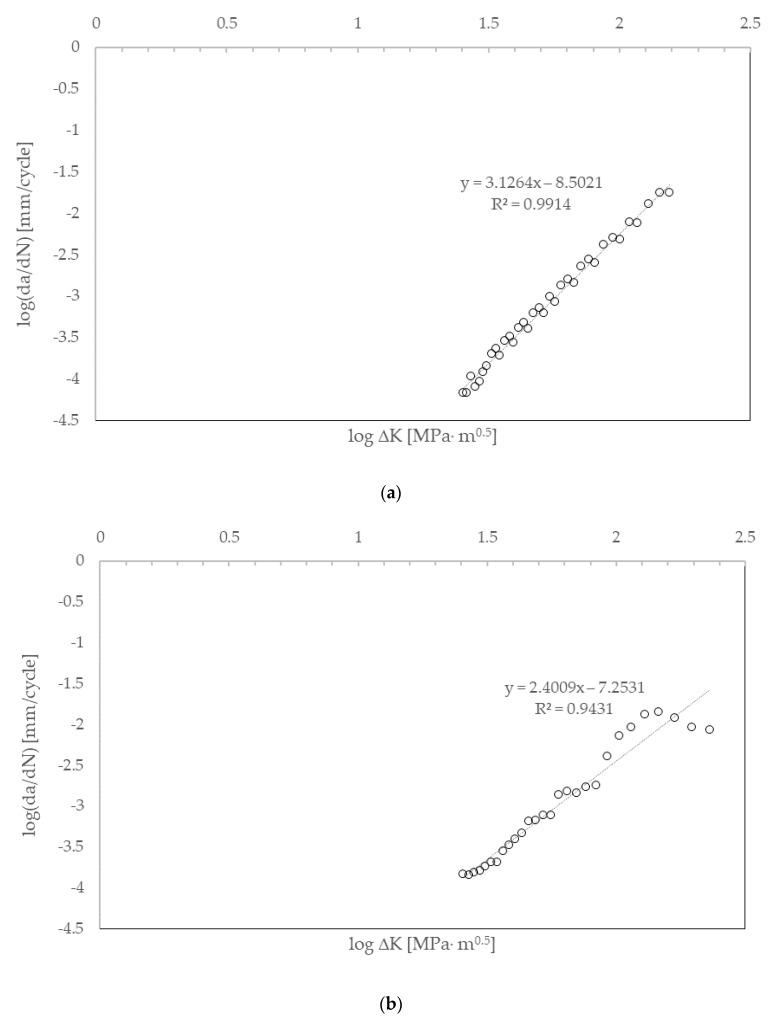
Crack propagation speed, *da*/*dN*, as a function of the stress intensity factor range, Δ*K*, (**a**) for the 55-element mesh along the propagation length; (**b**) for the 40-element mesh along the propagation length.

Fatigue crack propagation, crack length versus number of cycles, was simulated under mode I loading, considering two different refinements for the finite element meshes generated ahead of the pre-crack (Figure 6). The first mesh, with 55 elements placed along the crack propagation direction (*x*-axis), had a finite element edge length of 0.364 mm; the second mesh, not so refined, had 40 finite elements and a finite element edge length of 0.5 mm. Sixteen finite elements were placed along the specimen’s thickness for both meshes. Thereafter, parameters *C* and *m* of the Paris Law were calculated for both cases (Figure 7 and Table 6).

Considering the numerical values obtained for the Paris Law constants (Table 6) and comparing them with the experimental values presented in Section 2 (Table 3), it can be concluded that the results obtained through the software simulation and for the model with the highest mesh refinement (55 elements) indicate good accuracy in the fatigue analysis by Abaqus^®^ and the possibility of simulating fatigue crack propagation adequately.

## 4. Conclusions

The study herein presented uses a standardized specimen geometry (CT specimen), for which the values of the stress intensity factor in mode I loading are well known, to validate the suitability of the XFEM method to determine those values. Thereafter, mixed-mode loading was applied to the same CT specimen geometry, and correspondent stress intensity factors were calculated; crack propagation direction (crack path) was also estimated, and, finally, crack growth under cyclic mode I loading was assessed.

Based on the bibliographic research conducted, it was possible to find studies similar to the one presented here, involving the XFEM method applied to different structures and mechanical components for calculating stress intensity factors, particularly in various types of welded joints and components subjected to fretting and corrosion. However, the determination of stress intensity factors associated with mixed loading and the numerical simulation of crack propagation is almost inexistent, particularly with regard to the CT specimen geometry considered in this investigation.

Considering the results presented throughout the manuscript, the following conclusions can be drawn:

- XFEM makes it possible to obtain the direction of crack propagation very effectively and has also proved to be suitable to assess crack growth determination;
- Considering a standard Compact Tension (CT) specimen and an applied mode I loading, the results obtained for K_I_ were very close to the analytical ones;
- K_I_, K_II_, and K_III_ values were also calculated for mixed-mode loading (mode I+II). It was not possible to compare the calculated values with similar ones published in the literature;
- In the CT specimen and for the mode I loading, either static or cyclic, bending at the crack tip will not occur. Therefore, the C3D8R eight-node solid element used is enough for the purpose and allows the reduction in computational time. This was confirmed by the good agreement between the analytical and numerical stress intensity factor values, K_I_, as shown in Figure 3b. In addition, as shown in Table 5, and as expected, the stress intensity factor K_I_ is almost constant at the crack tip along the five nodes located throughout the thickness for the three different crack lengths considered;
- However, when dealing with mixed-mode loading (mode I+II), bending at the crack tip may occur. Nevertheless, the C3D8R finite element used and the number of elements along the thickness (four elements and five nodes) still seem adequate and sensible to the variation in the stress intensity factor values along the thickness, as shown in Figure 5b,c;
- FCG simulation carried out in this investigation using the XFEM method allowed the estimation of crack growth curves of the function of the number of cycles, a vs. N, closely to the FCGR curves experimentally determined in CT specimens under mode I loading. Moreover, slight differences in the Paris Law parameters, C and m, estimated were noticed in the function of the mesh refinement;
- These results prove the suitability of the method developed to predict the fatigue life of more complex mechanical components in which there will be an initial crack, provided that the parameters of the material’s Paris Law are appropriate, as well as the loading values and boundary conditions of the engineering problem. This will make it possible to define the times between periodical inspections more rigorously and thus guarantee the structural integrity of the components, avoiding their unexpected and catastrophic failure. The simulation of fatigue crack growth in engineering components will be addressed in future works.

## Figures and Tables

**Table 1:** Chemical composition of AISI 316L austenitic stainless steel [20,21].

	C (%)	Mn (%)	Cr (%)	Ni (%)	Mo (%)	N (%)	Si (%)	Cu (%)	V (%)	P (%)	S (%)
AISI 316L	<0.05	1.30	17.34	11.11	2.23	0.08	0.37	0.22	0.07	0.03	0.004

**Table 2:** Mechanical properties of the AISI 316L stainless steel at room temperature [20,21].

Yield Strength [MPa]	Tensile Strength [MPa]	Elongation [%]	Young’s Modulus, E [GPa]	Poisson Coefficient	Fracture Toughness [MPa·m^0.5^]
290	600	>50	200	0.3	100

**Table 3:** Experimental fatigue crack growth parameters obtained for AISI 316L at room temperature. Load ratio, R, equal to 0.1 [21].

Coefficient of the Paris Law, *C* [mm/Cycle; MPa·m^0.5^]	Exponent of the Paris Law, *m*	Fracture Toughness, *K_IC_* [MPa·m^0.5^]
1.04 × 10^−9^	3.34	100

**Table 4:** Analytical and numerical stress intensity factor values, K_I_, with deviation.

Crack Length, a [mm]	K_I_ Analytical [MPa·m^0.5^]	K_I_ Numerical [MPa·m^0.5^]	$\mathrm{Deviation}\;\left(\frac{KINumerical-KIAnalytical}{KIAnalytical}\right)$[%]
7.5	27.1	27.4	1.4
8.5	29.9	30.4	1.7
9.5	32.9	33.8	2.4
10.5	36.5	35.7	−2.2
11.5	40.5	40.3	−0.5
12.5	45.2	46.4	2.5
13.5	50.9	50.2	−1.4
14.5	57.9	57.8	−0.2
15.5	66.7	67.9	1.8
16.5	78.1	77.8	−0.4

**Table 5:** Average values of numerical stress intensity factor, K_I_ (5 contours), across the specimen’s thickness (nodes 1 to 5) for crack length, a, equal to 13.5 mm, 14.5 mm, and 15.5 mm.

Crack Length, *a* [mm]	K_I_ Numerical [MPa·m^0.5^]				
	Node 1	Node 2	Node 3	Node 4	Node 5
13.5	50.3	50.9	51	50.6	50.3
14.5	57	57.4	57.8	57.5	57.0
15.5	67.3	67.6	67.9	67.8	67.3

**Table 6:** Paris Law parameters obtained from the numerical simulations.

Mesh	Coefficient of the Paris Law, *C* [mm/Cycle; MPa·m^0.5^]	Exponent of the Paris Law, *m*
55 elements	3.147 × 10^−9^	3.13
40 elements	5.58 × 10^−8^	2.4

## Data Availability

The original contributions presented in the study are included in the article, further inquiries can be directed to the corresponding authors.

## Nomenclature

The following nomenclature are used in this manuscript:

*P*	Applied load
*B*	Specimen thickness
*W*	Effective width
*a*	Crack length
*E*	Young’s Modulus
K	Stress intensity factor
G	Energy release rate
N	Number of loading cycles
R	Load ratio in fatigue crack growth simulation

## References

[1] (2002). Standard Terminology Relating to Fatigue and Fracture Testing.

[2] Anderson T.L. (2017). Fracture Mechanics: Fundamentals and Applications.

[3] Pook L.P. (2015). The linear elastic analysis of cracked bodies and crack paths. Theor. Appl. Fract. Mech..

[4] Richard H.A., Fulland M., Sander M. (2004). Theoretical crack path prediction. Fatigue Fract. Eng. Mater. Struct..

[5] Zhu H. (2016). Mechanism of fatigue crack growth of bridge steel structures. Arch. Civ. Eng..

[6] Mutoh Y., Xu J., Kondoh K., Mutoh Y., Hoeppner D., Kinyon S. (2003). Observations and Analysis of Fretting Fatigue Crack Initiation and Propagation. Fretting Fatigue: Advances in Basic Understanding and Applications.

[7] He W., Liu J., Xie D. (2014). Numerical study on fatigue crack growth at a web-stiffener of ship structural details by an objected-oriented approach in conjunction with ABAQUS. Mar. Struct..

[8] Jie Z., Wang K., Liang S. (2022). Residual stress influence on fatigue crack propagation of CFRP strengthened welded joints. J. Constr. Steel Res..

[9] Da Silva A.L., Correia J.A., De Jesus A.M., Lesiuk G., Fernandes A.A., Calçada R., Berto F. (2019). Influence of fillet end geometry on fatigue behaviour of welded joints. Int. J. Fatigue.

[10] Huang C., Chen T., Xia Z., Jiang L. (2022). Numerical study of surface fatigue crack growth in steel plates repaired with CFRP. Eng. Struct..

[11] Tan H., Hu X., Wu X., Zeng Y., Tu X., Xu X., Qian J. (2021). Initial crack propagation of integral joint in steel truss arch bridges and its fatigue life accession. Eng. Fail. Anal..

[12] Liu D., Li Y., Xie X., Zhao J. (2019). Effect of Pre-Corrosion Pits on Residual Fatigue Life for 42CrMo Steel. Materials.

[13] Mohamed H.S., Gao F., Zhu H.P. (2018). The Influence of Crack Propagation Angle on the Stress Intensity Factors (SIFs) of Cracked Tubular T-Joints. Int. J. Steel Struct.

[14] Taleb W., Gardin C., Sarrazin-Baudoux C. (2021). 3D predictions of the local effective stress intensity factor as the fatigue crack propagation driving force. Int. J. Fatigue.

[15] Wang G., Ma Y., Guo Z., Bian H., Wang L., Zhang J. (2022). Fatigue life assessment of high-strength steel wires: Beach marks test and numerical investigation. Constr. Build. Mater..

[16] Leitner M., Simunek D., Shah S.F., Stoschka M. (2018). Numerical fatigue assessment of welded and HFMI-treated joints by notch stress/strain and fracture mechanical approaches. Adv. Eng. Softw..

[17] Poursaeidi E., Bazvandi H. (2016). Effects of emergency and fired shut down on transient thermal fatigue life of a gas turbine casing. Appl. Therm. Eng..

[18] Nie D., Chen X., Wu Q., Liu Y. (2020). Stress corrosion cracking behaviors of FV520B stainless steel used in a failed compressor impeller. Eng. Fail. Anal..

[19] Wang J., Gao Y. (2019). The stress intensity factor calculation for combined sliding wear and fatigue of GH4169 superalloy based on three-dimensional simulation. Wear.

[20] Martins R.F., Ferreira L., Reis L., Chambel P. (2016). Fatigue crack growth under cyclic torsional loading. Theor. Appl. Fract. Mech..

[21] Martins R.F., Branco C.M. (2004). A fatigue and creep study in austenitic stainless steel 316L used in exhaust pipes of naval gas turbines. Fatigue Fract. Eng. Mater. Struct..

[22] (2000). Standard Test Method for Measurement of Fatigue Crack Growth Rates.

[23] Benzeggagh M.L., Kenane M. (1996). Measurement of mixed-mode delamination fracture toughness of unidirectional glass/epoxy composites with mixed-mode bending apparatus. Compos. Sci. Technol..

[24] SIMULIA (2012). Abaqus Analysis User’s Guide.

